# Surgical and Clinical Aspects Associated with Double-Valve Infective Endocarditis

**DOI:** 10.3390/jcm14155589

**Published:** 2025-08-07

**Authors:** Sonia Lerta, Gloria Sangaletti, Vincenzo Antonio Villano, Flavia Puci, Eraldo Kushta, Pasquale Totaro, Filippo Amoroso, Giulia Magrini, Pietro Valsecchi, Raffaele Bruno, Elena Seminari

**Affiliations:** 1 Department of Medicine, Surgical, Diagnostic and Pediatric Science, University of Pavia, 27100 Pavia, Italy; sonia.lerta01@universitadipavia.it (S.L.); vincenzoantoni.villano01@universitadipavia.it (V.A.V.); flavia.puci01@universitadipavia.it (F.P.); eraldo.kushta01@universitadipavia.it (E.K.); raffaele.bruno@unipv.it (R.B.); 2 Infectious Diseases Unit, IRCCS Policlinico San Matteo, 27100 Pavia, Italy; p.valsecchi@smatteo.pv.it (P.V.); e.seminari@smatteo.pv.it (E.S.); 3 Cardiac Surgery Unit, IRCCS Policlinico San Matteo, 27100 Pavia, Italy; p.totaro@smatteo.pv.it (P.T.); filamoroso@hotmail.it (F.A.); 4 Division of Cardiology, IRCCS Policlinico San Matteo, 27100 Pavia, Italy; g.magrini@smatteo.pv.it

**Keywords:** double-valve infective endocarditis, surgical treatment, diagnostic strategies, in-hospital mortality, elderly patients, prognosis, observational study

## Abstract

**Background**: Double-valve infective endocarditis (DVIE) accounts for 15–20% of all endocarditis and represents a challenge due to the increased incidence of embolic events and congestive heart failure compared to infective endocarditis (IE) affecting one valve. This study aims to evaluate patients’ characteristics, surgical procedures, complications, and mortality associated with DVIE in our tertiary hospital in Italy. The Endocarditis Registry STEADY includes patients admitted with IE from January 2009 to March 2024 (*n* = 398). Sixty-three of them (16%) had DVIE. **Methods**: We conducted a retrospective single-center observational study, analyzing demographic, clinical, and microbiological data in DVIE patients, comparing those treated surgically (surgical group, SG) with those treated medically (non-surgical group, NSG). **Results**: The groups were homogeneous in age, microbiological yields, type of valve involved, and risk factors for infective endocarditis. The surgical group presented significantly more cancer history, intracardiac complications, and new-onset arrhythmias compared to the non-surgical group. Median hospital stay was similar in both groups. In SG, the most common postoperative complication was new rhythm disorders; other complications such as cardiac tamponade, pericardial effusion, and pneumothorax were rare. In-hospital mortality was similar between groups; however, one-year survival was higher in the surgical group (72% vs. 54%, *p* = 0.031). In our series, 16 patients were over 75 years old (25%), and 7 of them (44%) underwent cardiac surgery. One-year survival in the surgical group was also higher in this subgroup. **Conclusions**: Surgical treatment, when indicated, may improve the prognosis of patients with DVIE, including elderly patients.

## 1. Introduction

Infective endocarditis (IE) is a life-threatening condition characterized by high mortality and a high incidence of severe complications [1].

The epidemiology of IE has changed significantly over the years, and its incidence is increasing, partly due to an increase in staphylococcal and healthcare-associated IE, as well as to the emergence of new predisposing conditions such as the presence of prosthetic heart valves or intracardiac devices, hemodialysis, immunodeficiency, increased use of injectable treatments, and, most importantly, the aging of the population. These factors have changed the demographic and clinical profile of IE, making its prevention and management increasingly complex [2,3]. Multivalvular IE (MIE) is relatively uncommon, with an incidence ranging from 12% to 30% [4,5,6]. Specifically, double-valve infective endocarditis (DVIE) accounts for 15–20% of all endocarditis [7].

The mechanisms by which infection extends to involve two valves differ between left-sided and bilateral infective endocarditis. In left-sided DVIE, the mitral lesion typically occurs as a consequence of primary aortic endocarditis. The most common mechanism that leads to mitral valve involvement is the formation of a “jet lesion” caused by aortic regurgitation. The regurgitant jet may be directed to the mitral valve, leading to vegetation formation, leaflet perforation, or pseudoaneurysm of the anterior mitral leaflet. Another common mechanism is the one known as “kissing valves” in which the vegetation on the aortic valve extends towards the mitral valve, touching it and compromising it. In contrast, bilateral endocarditis often results from the hematogenous spread of the infection [2,6,7,8].

Surgery improves the outcome of IE, with lower risk of short- and long-term mortality [9]. Recent studies, including the work by Miller et al. [10], have reported improved survival rates in patients with DVIE in recent years, particularly among those undergoing surgery. These outcomes may be attributed to advancements in intraoperative and postoperative management. In-hospital associated mortality for DVIE after surgery ranges from 7% to 16% in published series [10,11,12]. Risk factors affecting mortality are represented by prosthetic valve endocarditis, requiring IABP (intra-aortic balloon pump) post-surgery, preoperative dialysis, and tricuspid involvement [10].

To improve prognosis, it is crucial to identify patients at the highest risk of complications as an aggressive therapeutic approach is likely to be beneficial for those at greater risk [3,10]. Moreover, few data are reported in the literature about outcomes in elderly patients, in particular in the setting of DVIE [13,14]. This gap is critical, as the aging population is increasingly affected by infective endocarditis, and double-valve involvement presents additional challenges in terms of management and prognosis [15].

In this study, we present the characteristics, prognosis, surgical procedures and their complications, and outcomes of patients with double-valve endocarditis treated at our tertiary hospital in Italy.

## 2. Materials and Methods

### 2.1. Inclusion Criteria

A retrospective observational study, based on records from the Fondazione IRCCS Policlinico San Matteo Endocarditis Registry “STEADY”, was conducted. Ethical approval was granted by the Ethics Committee of the Fondazione I.R.C.C.S. Policlinico “San Matteo” in Pavia (protocol number P-20200060). Adult patients admitted to IRCCS Policlinico San Matteo, in Pavia, Italy, from January 2009 to March 2024, with a diagnosis of infective endocarditis (IE) according to Duke’s criteria were included [16,17,18].

All cases were independently evaluated by an infectious disease specialist, a cardiologist, and a cardiothoracic surgeon before inclusion in the present study. Each patient underwent transthoracic echocardiography (TTE) and/or transesophageal echocardiography (TOE) as part of their diagnostic evaluation. A diagnosis of double-infective endocarditis (DVIE) was established when two valves were affected by an infectious process characterized by the presence of vegetation, valvular insufficiency, or both.

Data on demographic characteristics (age, gender, and other baseline characteristics), risk factors for endocarditis, comorbidities, site of infection, microbiology yield, clinical and laboratory features, complications of the infection, and outcome were collected.

Patients were divided into two groups: those undergoing cardiac surgery (surgical group, SG) and those who did not (non-surgical group, NSG). For patients undergoing surgical treatment, information about surgical procedures was also collected (type of procedure, type of valve implanted, complications, and other variables).

Surgical timing in our study was classified as non-urgent, urgent, and emergency according to the criteria of urgency outlined in the respective editions of the ESC Guidelines for the Management of Infective Endocarditis (2009, 2015, and 2023) that were current at the time of intervention [1,17,19]. EuroSCORE II was calculated for each surgical patient to estimate the probability of in-hospital mortality in patients undergoing cardiac surgery [20].

### 2.2. Definition of Primary and Secondary Endpoints

The primary endpoint was to describe the characteristics of DVIE in our tertiary hospital, comparing patients treated surgically (surgical group, SG) with those treated medically (non-surgical group, NSG), to assess any differences between those two groups in terms of demographic characteristics and outcomes.

The secondary endpoint focused on the patients who underwent surgery. The types of surgical procedures and their complications were registered.

### 2.3. Statistical Analysis

Concerning the primary endpoint of the study, categorical variables were expressed by count and percentage, while quantitative variables were described by median and IQR. Fisher’s exact test was used to compare the distribution of categorical variables in the two groups previously described (“surgical group” and “non-surgical group”), while the Wilcoxon rank sum test was used for quantitative variables for the two groups. *p*-values less than 0.05 were considered statistically significant.

For the secondary endpoint, we described the variables by count, percentage, median, and IQR. We also assessed factors associated with one-year survival in the surgical group using logistic regression models.

All analyses were performed using version 4.4.0 (version number 2024.09.1+394) of R Studio (R Foundation for Statistical Computing, Vienna, Austria).

## 3. Results

### 3.1. Baseline Characteristics

Of 398 patients with a diagnosis of infective endocarditis admitted in our hospital from January 2009 to March 2024, 63 patients (16%) had double-valve involvement; they were predominantly male (*n* = 52; 83%) with a median age at admission of 70 years (interquartile range [IQR] 57.00–74.50), with 16 patients (25%) being over 75 years old. Patients’ characteristics are summarized in Table 1.

**Figure 1:**
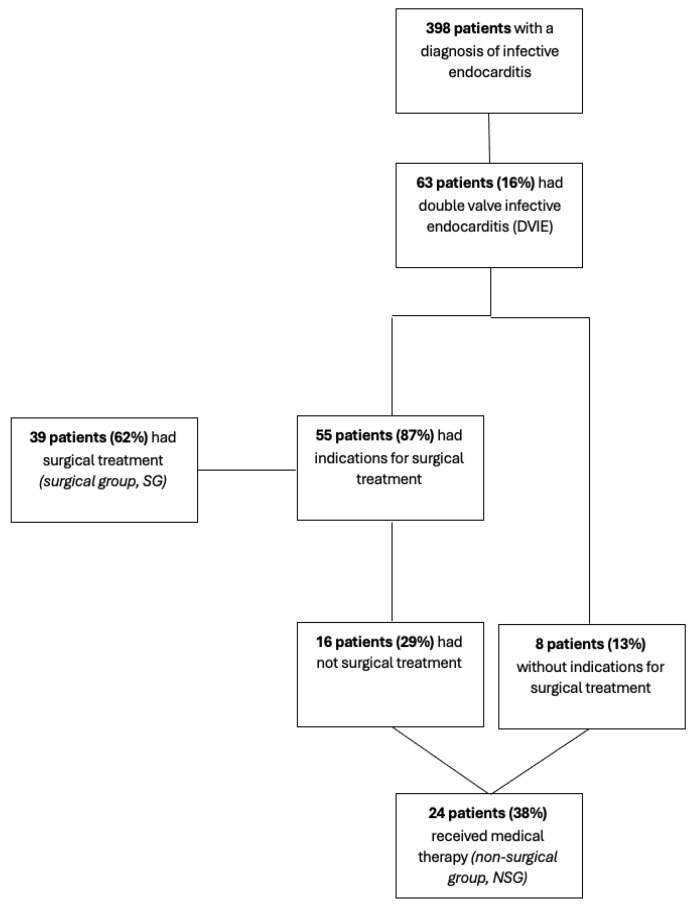
Distribution of patients.

Of the 55 patients (87%) who had indications for surgical treatment, 39 of them (62%) received surgery (surgical group, SG). The remaining 16 (29%) did not receive surgery. A total of 24 patients (38%) received medical therapy (non-surgical group, NSG). The distribution of patients among the treatment groups is illustrated in Figure 1.

### 3.2. Microbiology Yield

Two sets of blood cultures were performed for all patients upon admission to the hospital and were positive in 58 cases (92%). The most frequently identified pathogens were *Streptococcus* spp. (33%) and *Enterococci* (30%, mainly *Enterococcus faecalis*), followed by *Staphylococcus aureus* (13%) and coagulase-negative *Staphylococci* (9%). Six patients (7.9%) had culture-negative endocarditis.

Notably, the distribution of pathogens involved did not differ significantly between the surgical and non-surgical groups.

In the surgical group, valve cultures were performed in 35 cases (90%). Among these, five (14%) cases yielded positive results for the causative microorganism. The pathogens identified included *Staphylococcus epidermidis* (two cases) and one case each of *Enterococcus faecalis*, *Staphylococcus caprae*, and *Streptococcus gallolyticus*. The remaining valve cultures yielded negative results.

### 3.3. Risk Factors for IE

The risk factors for infective endocarditis of the study population were as follows: history of cardiovascular procedures (*n* = 17; 27%); anatomical alterations (*n* = 13; 21%) like aortic stenosis, mitral regurgitation, congenital alterations, dilatative cardiomyopathy; presence of PM or ICD at admission (*n* = 9; 14%); gastrointestinal alterations (*n* = 8; 13%); intravenous drug abuse (*n* = 8; 13%); recent dental procedures (*n* = 1; 1.6%); rheumatic fever (*n* = 1; 1.6%); skin lesions (*n* = 1; 1.6%).

Predisposing factors did not differ significantly in the two groups, except for a history of cardiovascular procedures, which was preponderant in the non-surgical group (*p* = 0.047).

### 3.4. Comorbidities

The main comorbidities of the patients in this study were represented by type II diabetes mellitus (*n* = 14; 22%), chronic hepatitis (*n* = 10; 16%), active neoplasia (*n* = 10; 16%), HIV (*n* = 4; 6%), and end-stage renal disease requiring hemodialysis (*n* = 2; 3.2%). Nine patients (14%) were treated with immunosuppressive therapy. Moreover, 22% (*n* = 14) were currently on anticoagulant therapy at admission, and 16% (*n* = 10) were on antiplatelet therapy.

The comorbidities did not differ significantly in the two groups, except for active neoplasia and immunosuppressive therapy being more frequent in the NSG (*p* = 0.034 and 0.021, respectively).

### 3.5. Echocardiography and Localization of IE

**Figure 2:**
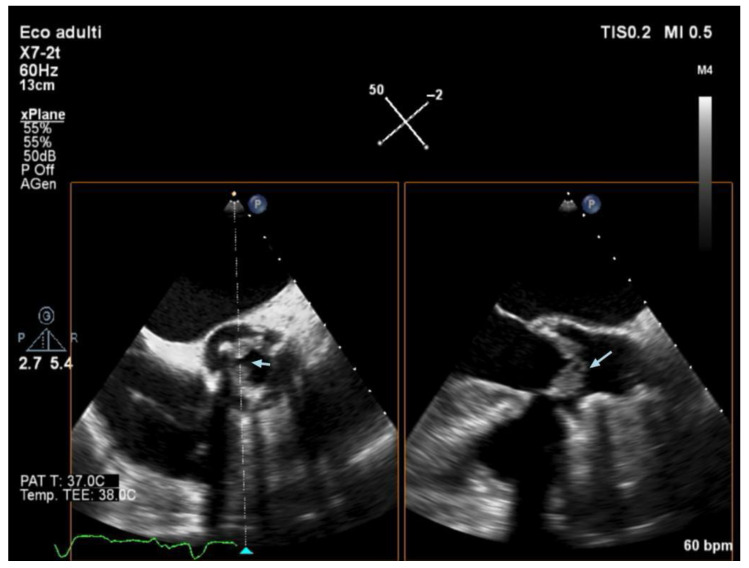
Transesophageal echocardiography—midesophageal aortic valve short axis (left) and long view (right) obtained with X-plane. Aortic valve bioprosthesis with evidence of irregular isohyporeflective thickening of the leaflets due to the presence of mobile vegetative lesions of an endocarditic nature.

**Figure 3:**
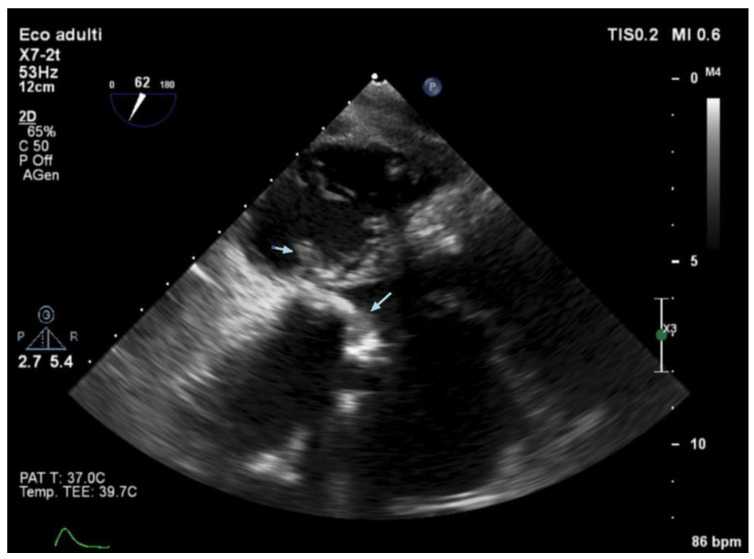
Transesophageal echocardiography—deep transgastric 5-chamber view. Tricuspid valve (left of image) showing a rounded, isohyporeflecting vegetative lesion (arrow) located between the septal leaflet and the anterior leaflet of the valve. Aortic bioprosthesis (right of image) showing diffuse, irregular leaflet thickening (arrow) due to the presence of mobile, endocarditic vegetative lesions.

**Figure 4:**
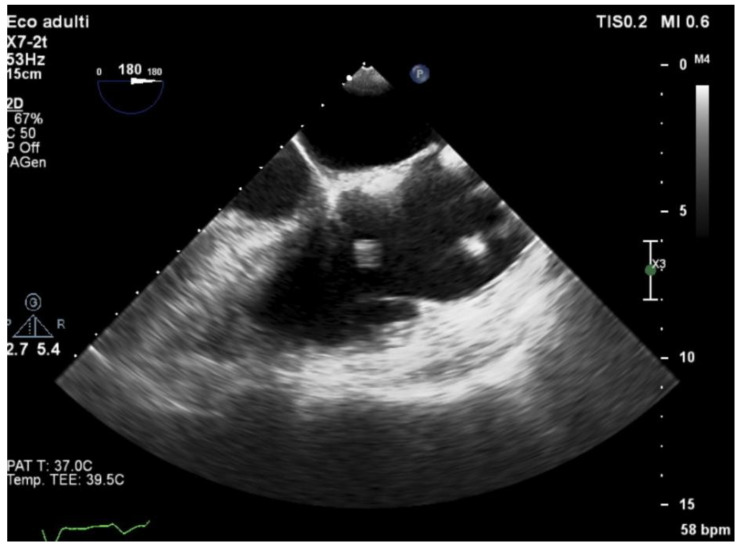
Transesophageal echocardiography—midesophageal RV-focused view: vegetating lesions attached to the right atrial portion of the pacemaker lead.

Echocardiography (TTE and/or TOE) was performed in all patients of our cohort and showed vegetations and/or severe valve dysfunction. Figure 2, Figure 3 and Figure 4 show examples of echocardiographic findings typical of DVIE.

The aortic and mitral valves (left endocarditis) were most involved (*n* = 50; 79%), followed by mitral and tricuspid valves (*n* = 6; 9.5%), aortic and tricuspid valves (*n* = 5; 7.9% of cases), and tricuspid and pulmonary valves (*n* = 2; 3.2%).

In 76% of cases (*n* = 48), the valves involved were both native; in 19% of cases (*n* = 12), one valve was native, and one was prosthetic; in 3.2% of cases (*n* = 2), both valves were prosthetic (Table 2). Of 14 patients with prosthetic valve(s), there was only one case of TAVI (Transcatheter Aortic Valve Implantation).

### 3.6. IE Complications

A complete list of the complications of infection in our series is shown in Table 1. The most frequent complications were septic emboli, found in nearly half of the patients, mainly cerebral (*n* = 20; 32%), without significant differences between the two groups.

Intracardiac complications, such as perivalvular abscess, pseudoaneurysm, paravalvular leak, and rupture of chordae tendineae, were found in 21 patients (33%), significantly more in the surgical group (*p* = 0.007). New-onset arrhythmias occurred in eight patients (13%); all of them belonged to the surgical group (*p* = 0.020).

### 3.7. Clinical Course and Treatment

Of 63 patients, 24 received medical therapy; meanwhile, 39 patients underwent cardiac surgery. The main indications for surgery were severe valvular regurgitation with NYHA ≥ III (*n* = 23; 59%), heart failure (*n* = 7; 18%), presence of vegetation > 1 cm (*n* = 4; 10%), infected prosthesis (*n* = 3; 7%), and presence of aortic abscess/aneurysm/pseudoaneurysm (*n* = 2; 5%).

The overall median duration of IV antibiotic therapy was 38 days (IQR 29–46.25): in the non-surgical group, the median duration of therapy was 32 days (IQR 29.50–41.50); in the surgical group, the median duration of therapy was 42 days (IQR 29.00–51.00). Eleven patients (17%) were able to switch to oral antibiotic therapy: five (21%) in the non-surgical group and six (15%) in the surgical group.

### 3.8. Surgical Procedures

Of 55 patients (87%) who had an indication of surgery, 39 (71%) underwent a cardiac surgical intervention. Their median EuroSCORE II (European System for Cardiac Operative Risk Evaluation) was 7.0 (IQR 4.0–8.0). The remaining 16 patients (29%) were excluded for high surgical risk and/or technical unfeasibility (14 patients: 2 of them had cachexia, 6 had active cancer) or patient refusal (2 patients). Notably, their median EuroSCORE II was 11.0 (IQR 9.0–13.0).

Among the 16 elderly patients (those over 75 years old, representing 25% of the cohort), 7 (44%) underwent cardiac surgery.

Surgical timing in our study was classified as non-urgent, urgent, and emergency according to the criteria of urgency outlined in the respective editions of the ESC Guidelines for the Management of Infective Endocarditis (2009, 2015, and 2023) that were current at the time of intervention [1,17,19]. Surgery was defined as non-urgent for patients with stable cardiac function days to weeks before the operation, urgent within 3–5 days for procedures required during the same hospitalization to minimize clinical deterioration, and emergent for cardiac compromise not responsive to any form of therapy except surgery. Eighteen patients (46%) were treated urgently, with a median of 4 (IQR 2.0–6.0) days between diagnosis of IE (and cardiac surgery consult) and surgical procedure. The primary reasons for urgent surgery were poor hemodynamic compensation (11 patients; 61%), local complications such as abscess or pseudoaneurysm (4 patients; 22%), and vegetations with high embolic potential (3 patients; 16%). The remaining 21 patients (54%) underwent non-urgent surgery. No patient underwent emergency surgery (within 24 h).

Native valvular endocarditis (NVE) was identified in 33 patients (85%), while prosthetic valvular endocarditis (PVE) involving a single valve was found in 6 patients (15%). Most patients presented mitral–aortic involvement (*n* = 29; 74%), followed by mitral–tricuspid involvement (*n* = 5; 13%), aortic–tricuspid involvement (*n* = 3; 7.7%), and two cases of tricuspid–pulmonary involvement (*n* = 2; 5.1%). Mitral repair was chosen as the first treatment option for 13 patients with mitral involvement. In cases where repair was not feasible, a prosthetic mitral valve was implanted in 21 cases (16 biological, 5 mechanical). Aortic valve surgery consisted in replacement in 30 cases (with biological prosthesis in 26 cases, mechanical prosthesis in 4 cases). Aortic valve repair was performed only in one case. A complete list of all the surgical procedures can be found in Table 3.

An aortic abscess was identified in three patients (8%) and was treated with aortic root replacement using an allograft (Freestyle™ Aortic Root Bioprosthesis, Medtronic Inc., Minneapolis, MN, USA). Additionally, an abscess of the fibrous skeleton was identified in six patients (15%) and was treated with reconstruction of the intervalvular fibrous body with a pericardial bovine patch. In three cases of mitro-aortic endocarditis, surgeons found tricuspidal involvement during the intervention and performed tricuspidal repair.

The median extracorporeal circulation time was 156 (IQR 121–191) minutes, and the median aortic cross-clamp time was 119 (IQR 85–153) minutes. Associated ascending aorta replacement was performed in two cases, and concomitant myocardial revascularization was required in eight cases. None of the patients needed postoperative extracorporeal assistance with ECMO or IABP.

The median intensive care unit (ICU) stay was 2 days (IQR 1–6), and the median postoperative hospital stay was 13 days (IQR 9–21). All patients were transferred to another facility for continued cardiopulmonary rehabilitation.

### 3.9. Postoperative Complications

Postoperative complications are summarized in Table 4. The most common complications were new-onset cardiac rhythm disorders (*n* = 16; 41%), including nine patients (23%) requiring pacemaker implantation.

There were two cases (5%) of cardiac tamponade, one case (2%) of paravalvular leak, one case (2%) of pneumothorax, and one case (2%) of pericardial effusion.

There were no cases of postoperative pneumonia, intestinal ischemia, or major neurological disorders. However, one patient developed acute renal injury requiring dialysis that was later discontinued after normalization of renal function.

### 3.10. Outcomes

The median total hospital length of stay was 36.50 days (IQR 26.25–50) overall, and it did not differ in the two groups.

The overall in-hospital mortality rate was 11% (21% in the non-surgical group and 5.1% in the surgical group), with no statistically significant differences between the two groups considered (*p*-value 0.095).

Among the sixteen elderly patients, the in-hospital mortality rate was 25% (*n* = 4). We recorded two deaths among elderly surgical patients (28%) and two among those treated with medical therapy alone (22%).

Meanwhile, one-year survival (defined as survival one year from the date of hospital admission) was 69% overall. Notably, one-year survival was higher in the surgical group (80% versus 54%, *p* = 0.046), including for patients over 75 years old (57% versus 22%). We used logistic regression to investigate the association between one-year survival and variables in the subgroup SG, without significant findings, as shown in Table 5.

## 4. Discussion

Our study aimed to compare the demographic characteristics and outcomes of patients with DVIE who underwent surgery versus those treated with medical therapy alone, with a particular focus on surgical procedures and their associated complications in the surgical group.

Double-valve infective endocarditis affects approximately 20% of patients with IE and is associated with an in-hospital mortality ranging from 14 to 34% [2,4,7,10]. In our cohort, DVIE accounts for approximately 16% of infective endocarditis cases, including native and prosthetic DVIE, and the most common pattern of DVIE involves aortic and mitral valves (79%) with a presumable jet lesion on the mitral valve. In 76% of cases, native valves were involved. In the study conducted by Scheggi et al., left-sided heart involvement was observed in 81% of cases, while in the work of Bohbot et al., it was observed in 70%. Regarding native valves, Scheggi et al. reported a prevalence of 79%, while Bohbot et al. recorded 75% [2,7]. These data suggest a predominance of left-sided heart involvement and native valves in DVIE.

Scheggi et al. [7], Bohbot et al. [2], and Selton-Suty et al. [6] reported that patients with multivalvular IE had a higher prevalence of streptococcal infection. Accordingly, the most common etiologic microorganism in our series was *Streptococcus* species (33%), followed by *Enterococci* (30%).

This study presents the clinical profile of 63 patients with double-valve infective endocarditis, of whom 39 underwent cardiac surgery, while 24 received medical therapy alone. All patients received intravenous antibiotic therapy for a median duration of 38 days (IQR 29–46.25). The in-hospital mortality rate was 11% overall. In-hospital mortality in surgically treated patients was 5.1% compared to 21% in those treated with medical therapy alone. Additionally, one-year survival was higher in the surgical group.

Pizzino et al. have identified IE-related heart failure and embolic events as independent predictors of major adverse events (death, recurrence of infection), in 102 patients with IE and surgical indication [21]. However, in our series, we did not find any variable associated with one-year survival in surgical patients, possibly due to the limited number of patients.

Even among elderly patients (considering patients over 75 years old), one-year survival was higher in the surgical group, with a survival rate of 75% compared to 22% in the non-surgical group. The current literature reveals a significant lack of data on outcomes in elderly patients, particularly in the context of DVIE. Older patients often present with comorbidities, which can substantially impact their response to treatment and increase the risk of surgical complications. In a recent issue of the *Journal of the American Heart Association* (JAHA), Ragnarsson and colleagues provide an analysis that evaluates surgical use and outcomes stratified by age [13]. Although older age is a known risk factor, cardiac surgery outcomes in elderly IE patients who meet surgical indications have shown excellent survival rates and quality of life following the procedure. Furthermore, elderly patients are increasingly afflicted with IE and should not be denied surgery based on age alone. Shared decision-making and experienced multidisciplinary teams are required for best evidence-based practice in these complex patients. In contrast to their analysis, where 69% of patients met the surgical indication, only 51% underwent surgery, mainly due to patient refusal of high-risk surgery, in our group of patients, 44% had a surgical indication, and all underwent surgery. This highlights the importance of a multidisciplinary team in the evaluation and management of patients [13,14,22].

Previous studies have shown the positive impact of early surgical intervention for infective endocarditis on both early and late survival. American Heart Association guidelines suggest that early surgery, combined with antibiotic therapy, is appropriate in approximately 50% of IE cases to properly manage the infection. Reducing the duration of infection may be associated with a reduced risk of embolization or long-term myocardial dysfunction associated with prolonged valvular damage and inflammation [23].

The study conducted by Miller et al. compares the therapeutic approach to DVIE over two decades (2001–2010 and 2011–2021) and demonstrates an improvement in medium- to long-term survival in the last decade among patients affected by this disease and managed surgically. This is associated with earlier hospital presentation and, consequently, early evaluation [10]. Multidisciplinary care has been established and implemented for valvular and structural heart diseases, and its application to infective endocarditis, including IE after structural heart interventions [24], has developed recently with the growth of endocarditis teams at multiple institutions [10]. IE is a complex disease, in which several factors, such as underlying cardiac disorders and pre-existing comorbidities, extent of cardiac involvement by infection, systemic spread of the disease with/without multi-organ involvement, the type of causal microorganisms, and presence or absence of sepsis or septic shock and/or cardiomyopathy, play an important role in tailoring the management for every patient [17]. Especially considering the current diagnostic challenges in IE diagnosis (e.g., the increasing role of prosthetic valves and TAVI, where detecting echocardiographic signs of IE can be more difficult, and the need for other imaging techniques such as PET-CT and cardio-synchronized CT), the role of an endocarditis team that includes cardiologists, cardiac surgeons, infectious diseases specialists, microbiologists, and radiologists is crucial. Early detection of the disease and management before development of complications such as heart failure, peri-annular abscesses, and/or embolic events are of utmost importance [25].

The risk of surgical treatment during the active phase of IE can be significant. It is heavily influenced by pre-existing comorbidities and current organ function but should not be limited by one risk factor alone. For each surgical patient, we calculated the EuroSCORE II to provide a more accurate prediction of in-hospital mortality by incorporating additional clinical variables. This tool guides decision-making regarding cardiac surgery and supports clinicians in counseling patients and their families about the associated surgical risks. There are three main reasons to undergo surgery in the setting of acute IE: heart failure, uncontrolled infection, and prevention of septic embolization (in particular, in the central nervous system) [20].

In our study, mitral repair was chosen as a first-line treatment option in 11 patients, reflecting the preference for valve preservation strategies where feasible. Repair techniques are generally preferred for their potential to preserve native valve function, reduce the risk of prosthesis-related complications, and avoid the need for long-term anticoagulation. However, the feasibility of repair depends heavily on the extent of valve damage, the underlying pathology, and patient-specific factors. In cases where the repair was not possible due to extensive structural damage or adverse anatomical conditions, replacement was required.

Among the 39 patients who were treated surgically, valve culture was performed in 35 (90%). Only five (14%) of those were positive, all for the same microorganisms found in blood cultures (*S. gallolyticus* in one case, *E. faecalis* in one case, *S. epidermidis* in two cases, *S. caprae* in one case). Some studies identify valve-culture positivity as a significant risk factor for poor outcomes. In Kim et al.’s series, 1-year mortality rate was significantly higher in patients with positive valve culture (31.8% vs. 8.9%, *p* = 0.005), while Garcia et al. identified positive valve culture (in patients who also had positive blood cultures) as an independent predictor of hospital mortality, finding nearly 2-fold higher in-hospital mortality compared to the patients with positive blood cultures and negative valve culture [26,27]. However, in our small series, in-hospital mortality in patients with positive valve culture was 0%, four out of five patients survived at least one year from admission, and one was lost to follow-up.

### Study Limitations

The current study is single-center and retrospective, thus presenting intrinsic limitations that must be considered, such as limited generalizability, as the data come from a single center and may not represent the general population. Moreover, the study was performed at a tertiary hospital; in fact, our sample does not reflect the characteristics of all patients with infective endocarditis in the general population, but rather the population of patients with endocarditis admitted to dedicated hospitals. Accordingly, our conclusions apply to reference hospitals equipped and staffed to perform heart surgery.

The number of patients included might be lower compared to multicenter studies, reducing the statistical power.

Data were collected retrospectively from medical records, meaning that only patients with available data and confirmed diagnoses are included in the study, potentially excluding relevant cases that could influence the results.

## 5. Conclusions

In conclusion, double-valve infective endocarditis (DVIE) represents a considerable proportion of overall cases of infective endocarditis, with involvement of mitral and aortic valves being the most common. DVIE complicates surgical procedures, increasing embolic events and congestive heart failure. DVIE has an adverse prognosis due to its high rate of complications.

The evaluation of surgical outcomes in the patients of this study showed positive results. When indicated, surgical treatment can improve patient prognosis, including in elderly patients. However, there are few studies in the literature on these aspects, and even fewer with long-term follow-up.

Multidisciplinary evaluation for DVIE may be considered to better understand the optimal timing and repair strategy and is associated with better outcomes in infective endocarditis.

## Figures and Tables

**Table 1:** Patients’ characteristics, pathogens, valve culture performed and positive, localization, type of valve, risk factors for IE, comorbidities, complications of infection, EV antibiotic duration, switch to oral antibiotics, in-hospital mortality, one-year survival.

	All	Groups		
Characteristics	*N* = 63	NSG ^1^, *N* = 24	SG ^1^, *N* = 39	*p*-Value ^2^
Male sex, *n* (%)	52 (83%)	18 (75%)	34 (87%)	0.3
Age, median (IQ)	70.00 (17.50)	71.50 (20.00)	68.00 (15.50)	0.3
Age > 75 years old, *n* (%)	16 (25%)	9 (56%)	7 (44%)	0.08
Length of hospital stay, median (IQ)	36.50 (23.75)	30.50 (23.50)	37.50 (20.25)	0.2
**Pathogens, *n* (%)**				0.3
Coagulase-negative Staphylococci	6 (9.5%)	1 (4.2%)	5 (13%)	
Enterococci	19 (30%)	9 (38%)	10 (26%)	
Negative	5 (7.9%)	0 (0%)	5 (13%)	
Others	4 (6.3%)	1 (4.2%)	3 (7.7%)	
*Staphylococcus aureus*	8 (13%)	4 (17%)	4 (10%)	
*Streptococcus* spp.	21 (33%)	9 (38%)	12 (31%)	
Time to negative blood cultures, days (IQR)	8.00 (9.25)	9.50 (9.25)	8.00 (7.00)	0.7
Valve culture performed, *n* (%)	/	/	35 (90%)	
Valve culture positive, *n* (%)	/	/	5 (14%)	
**Localization, *n* (%)**				0.6
AM [aortic–mitral]	50 (79%)	21 (88%)	29 (74%)	
AT [aortic–tricuspidal]	5 (7.9%)	2 (8.3%)	3 (7.7%)	
MT [mitral–tricuspidal]	6 (9.5%)	1 (4.2%)	5 (13%)	
TP [tricuspidal–pulmonary]	2 (3.2%)	0 (0%)	2 (5.1%)	
**Type of valve, *n* (%)**				0.050
NN [native–native]	48 (76%)	15 (63%)	33 (85%)	
NP [native–prosthetic]	12 (19%)	6 (25%)	6 (15%)	
PP [prosthetic–prosthetic]	2 (3.2%)	2 (8.3%)	0 (0%)	
Unknown	1 (1.6%)	1 (4.2%)	0 (0%)	
**Risk factors for IE, *n* (%)**				
Cardiovascular procedure history	17 (27%)	10 (42%)	7 (18%)	0.047
ICD/PM at admission	9 (14%)	2 (8.3%)	7 (18%)	0.5
Intravenous drug use	8 (13%)	3 (13%)	5 (13%)	>0.9
Anatomical alterations	13 (21%)	7 (29%)	6 (15%)	0.2
Skin lesions	1 (1.6%)	1 (4.2%)	0 (0%)	0.4
Gastrointestinal alterations	8 (13%)	4 (17%)	4 (10%)	0.5
History of rheumatic fever	1 (1.6%)	0 (0%)	1 (2.6%)	>0.9
Recent dental procedures	1 (1.6%)	0 (0%)	1 (2.6%)	>0.9
**Comorbidities, *n* (%)**				
Chronic hepatitis	10 (16%)	4 (17%)	6 (15%)	>0.9
Hemodialysis	2 (3.2%)	2 (8.3%)	0 (0%)	0.14
Type 2 diabetes	14 (22%)	8 (33%)	6 (15%)	0.12
HIV	4 (6.3%)	0 (0%)	4 (10%)	0.3
Cancer	10 (16%)	7 (29%)	3 (7.7%)	0.034
Immunosuppressive therapy	9 (14%)	7 (29%)	2 (5.1%)	0.021
Anticoagulant therapy	14 (22%)	8 (33%)	6 (15%)	0.12
Antiplatelet therapy	10 (16%)	6 (25%)	4 (10%)	0.2
**Complications of infection, *n* (%)**				
Cardiac complications	21 (33%)	3 (13%)	18 (46%)	0.007
Embolic complications	31 (49%)	9 (38%)	22 (56%)	0.2
Cerebral emboli	20 (32%)	6 (25%)	14 (36%)	0.4
Spleen/kidney emboli	12 (19%)	3 (13%)	9 (23%)	0.3
Cutaneous emboli	2 (3.2%)	1 (4.2%)	1 (2.6%)	>0.9
Spondylodiscitis	7 (11%)	1 (4.2%)	6 (15%)	0.2
Pulmonary emboli	7 (11%)	2 (8.3%)	5 (13%)	0.7
Septic shock	2 (3.2%)	1 (4.2%)	1 (2.6%)	>0.9
Arrhythmias	8 (13%)	0 (0%)	8 (21%)	0.020
**EV antibiotic duration, days (IQ)**	38.00 (17.25)	32.00 (12.00)	42.00 (22.00)	0.12
**Switch to oral antibiotics, *n* (%)**	11 (17%)	5 (21%)	6 (15%)	0.7
**In-hospital mortality, *n* (%)**	7 (11%)	5 (21%)	2 (5.1%)	0.095
**One-year survival, *n* (%)** Unknown, *n* (%)	41 (65%) 4 (6.3%)	13 (54%) 0 (0%)	28 (72%) 4 (10%)	0.031

^1^ NSG = non-surgical group; SG = surgical group; ^2^ Fisher’s exact test, Wilcoxon rank sum test.

**Table 2:** Native valves versus prosthetic valve(s).

	Total	Groups		
Characteristics	*N* = 63	Native Valves *N* = 49 (77.8%)	Prosthetic Valve(s) *N* = 14 (22.2%)	*p*-Value ^1^
Male sex, *n* (%)	52 (82.5%)	40 (81.6%)	12 (85.7%)	1.000
Age, median (IQR)	70.0 (57.0–74.5)	65.0 (56.0–74.0)	71.5 (69.2–77.0)	0.053
Length of hospital stay, days, median (IQR)	36.5 (26.2–50.0)	34.5 (26.0–50.0)	38.5 (31.2–45.5)	0.923
One-year survival	41 (65.1%)	37 (75.5%)	4 (28.6%)	0.003
Emboli	31 (49.2%)	24 (49.0%)	7 (50.0%)	1.000
Cerebral emboli	20 (31.7%)	16 (32.7%)	4 (28.6%)	1.000
Spleen or kidney emboli	12 (19.0%)	10 (20.4%)	2 (14.3%)	0.898
Skin emboli	1 (1.6%)	1 (2.0%)	0 (0.0%)	1.000
Osteomyelitis	7 (11.1%)	5 (10.2%)	2 (14.3%)	1.000
Pulmonary emboli	7 (11.1%)	6 (12.2%)	1 (7.1%)	0.957
Surgically treated	39 (61.9%)	33 (67.3%)	6 (42.9%)	0.176
In-hospital mortality	7 (11.1%)	3 (6.8%)	4 (28.6%)	0.052

^1^ Fisher’s exact test, Wilcoxon rank sum test.

**Table 3:** Surgical procedures.

Localization	Surgical Procedure	No.
Aortic–mitral [AM] (*n* = 29)	Double prosthetic valve Aortic prosthesis + mitral repair Mitral prosthesis + aortic repair Mitral prosthesis + tricuspidal repair	16 (14 biological, 2 mechanical) 11 (9 biological, 2 mechanical) 1 (mechanical) 1 (mechanical)
Mitral–tricuspidal [MT] (*n* = 5)	Mitral prosthesis + tricuspidal repair Tricuspid prosthesis + mitral repair Double repair	2 (1 mechanical) 1 (biological) 2
Aortic–tricuspidal [AT] (*n* = 3)	Aortic prosthesis + tricuspidal repair	3 (biological)
Tricuspidal–pulmonary [TP] (*n* = 2)	Tricuspid prosthesis + pulmonary valve repair Pulmonary prosthesis + tricuspidal repair	1 (biological) 1 (biological)
Additional procedures	CABG Ascending aorta replacement Aortic root replacement	8 (23%) 2 (6%) 1 (3%)

**Table 4:** Surgical variables and complications.

Characteristics of SG	*N* = 39
ICU stay, days (IQ) Cardiac surgery department stay, days (IQ) In-hospital mortality, *n* (%)	2.00 (5.00) 8.00 (4.75) 2 (5%)
Complications, *n* (%) AVB/Arrhythmias Post-op PM implantation Periprosthetic leak PNX Cardiac tamponade Pleural effusion Post-op dialysis	16 (41%) 9 (25%) 1 (2.6%) 1 (2.6%) 2 (5.1%) 1 (2.6%) 1 (2.6%)
EuroSCORE II, median (IQ) ECC time, minutes (IQ) Aortic cross-clamping time, minutes (IQ)	7.00 (4.00) 156.00 (70.00) 119.00 (68.25)
Timing of surgery, *n* (%) Non urgent Urgent	21 (54%) 18 (46%)

**Table 5:** Univariate logistic regression of features possibly associated with one-year survival (dependent variable) in surgical patients.

Variable	OR	IC 95%	*p*-Value
Cerebral emboli	0.632	0.116–3.44	0.595
Cardiac complications	2.17	0.358–13.11	0.400
Prosthetic valve	0.300	0.0394–2.29	0.245
Age (by 1 year)	0.957	0.879–1.04	0.957
Spleen/kidney emboli	1.64	0.164–16.34	0.675
Urgent surgery	0.222	0.0363–1.36	0.104

## Data Availability

The data that support the findings of this study are available from the corresponding author [GS], upon reasonable request.
